# Clinical Profile of Chinese Long-Term Parkinson’s Disease Survivors With 10 Years of Disease Duration and Beyond

**DOI:** 10.14336/AD.2017.0204

**Published:** 2018-02-01

**Authors:** Qian Sun, Tian Wang, Tian-Fang Jiang, Pei Huang, Ying Wang, Qin Xiao, Jun Liu, Sheng-Di Chen

**Affiliations:** Department of Neurology & Collaborative Innovation Center for Brain Science, Ruijin Hospital affiliated to Shanghai Jiao Tong University School of Medicine, Shanghai, 200025 China; Department of Neurology & Collaborative Innovation Center for Brain Science, Ruijin Hospital affiliated to Shanghai Jiao Tong University School of Medicine, Shanghai, 200025 China; Department of Neurology & Collaborative Innovation Center for Brain Science, Ruijin Hospital affiliated to Shanghai Jiao Tong University School of Medicine, Shanghai, 200025 China; Department of Neurology & Collaborative Innovation Center for Brain Science, Ruijin Hospital affiliated to Shanghai Jiao Tong University School of Medicine, Shanghai, 200025 China; Department of Neurology & Collaborative Innovation Center for Brain Science, Ruijin Hospital affiliated to Shanghai Jiao Tong University School of Medicine, Shanghai, 200025 China; Department of Neurology & Collaborative Innovation Center for Brain Science, Ruijin Hospital affiliated to Shanghai Jiao Tong University School of Medicine, Shanghai, 200025 China; Department of Neurology & Collaborative Innovation Center for Brain Science, Ruijin Hospital affiliated to Shanghai Jiao Tong University School of Medicine, Shanghai, 200025 China; Department of Neurology & Collaborative Innovation Center for Brain Science, Ruijin Hospital affiliated to Shanghai Jiao Tong University School of Medicine, Shanghai, 200025 China

**Keywords:** Parkinson’s disease, longevity, motor and non-motor symptoms, quality of life

## Abstract

**Background:**

Parkinson’s disease (PD) patients with 10 years or more survival (PD-10) are not well characterized. The aim of this study was to evaluate the main issues facing PD-10 patients and identify factors that independently contributed to quality of life (QoL).

**Methods:**

A group of 121 PD-10 patients recruited from outpatient clinics participated in this cross-sectional study. Data on demographic and clinical factors were collected. Multiple linear regression analyses were conducted to identify determinants of poor QoL.

**Results:**

The entire PD-10 patients had disease duration ranging from 10 to 23 years, with 84.2% of the total cohort skewed to between 10 and 15 years’ duration. The PD-10 patients had great frequency of left-sided onset, increased motor and non-motor symptoms as well as inferior QoL. The more advanced stage of disease in PD-10 patients was associated with motor phenotype, freezing of gait, higher UPDRS sub-scores and levodopa equivalent dose, less balanced confidence, fatigue, anxiety, depression, reduced quality of life and worse Timed Up & Go performance. Self-reported mood symptoms, decreased balance confidence and reduced daily activities were the three factors most closely associated with poorer QoL, but excessive daytime sleepiness and long disease duration additionally contributed to the explanatory power.

**Conclusions:**

This is the first report to investigate the clinical characteristics of Chinese PD-10 patients. Our study may elucidate an important clue for understanding PD-10 patients in clinical practice and identifying patients with PD at risk for reduced QoL.

Parkinson’s disease (PD) is the second most common neurodegenerative disease after Alzheimer’s disease. Clinically, it is characterized by resting tremor, rigidity, bradykinesia, and postural instability and a variety of poorly treatable non-motor symptoms such as fatigue, anxiety, depression, autonomic dysfunction, sleep disorders and cognitive deficits.

The available data suggests that by five years of PD duration the diagnosis is more reliable and as time progresses, PD patients begin to develop hallmark motor symptoms and increasing disability and mortality [1-3]. Additionally, the motor complications that include wearing off, random oscillations (on-off effect) and delayed "on" effect are also more prominent [4]. Over time, disease burden accumulates and the daily life functioning of patients declines.

To the best of our knowledge, there are a limited number of studies following longer disease duration PD patients [2, 3, 5-8]. Such information acquired from PD patients with 10-year disease duration and beyond will be invaluable in our understanding of the natural course of the disease and for health resources planning in an ageing society.

This study endeavored to identify the main issues facing patients when they reached the 10-year disease duration milestone. We chose 10 years as a point that demarcates clearly a diagnosis of true idiopathic PD firstly. Secondly, PD-10 patients may exhibit some unique clinical characteristics different from the early PD patients according to the literature [2, 3, 5-8]. Additionally, in the PD-10-time frame patients are usually medication dependent, and disease burdens are typically increased. The true nature of clinical characteristics and treatment strategies in Chinese PD-10 patients deserves further investigation.

With increased PD duration, there is an increased difficulty in maintaining a high quality of life (QoL). Compared with the general population, patients with PD reported lower levels of QoL in terms of physical, emotional well-being, and social functioning [9]. Mood symptoms and axial impairment were found to be the main consistent determinants of reduced QoL in PD [10]. A careful investigation of the data obtained from PD-10 cohort may give us important information about the main source of patients’ distress. Additionally, understanding how factors influence the subgroups of patients can help to individualize treatment strategies and relieve the disease burden.

Therefore, the purpose of this investigation was to identify the main issues facing PD patients with 10 years or more survival, and to determine factors that independently contribute to QoL.

## MATERIALS AND METHODS

### Subjects

In this cross-sectional study, all the PD patients with 10 years of disease duration and beyond were recruited between September 2014 and August 2016 from the Movement Disorder Specialist Clinic at the Department of Neurology, Ruijin Hospital affiliated to Shanghai Jiao Tong University School of Medicine, Shanghai, China. PD diagnosis was carried out using United Kingdom PD Society Brain Bank criteria [11]. PD patients who were treated with deep brain stimulation and continuous infusion of Levodopa Carbidopa Intestinal Gel were excluded. All the participants provided written informed consent, and the study was approved by the Research Ethics Committee, Ruijin Hospital affiliated to Shanghai Jiao Tong University School of Medicine, Shanghai, China.

### Subject evaluation

The following demographic and clinical data were collected from all patients: gender, age, age at onset, disease duration, motor asymmetry and medical history. Patient evaluation was carried out by movement disorder specialists. Disease duration was defined as time since the first motor symptom onset. Disease severity was evaluated by the Unified Parkinson’s Disease Rating Scale (UPDRS) [12] and the modified Hoehn and Yahr (H-Y) scale [13] while patients were on treatment. As the occurrence of motor complications was an important problem in the long-term dopaminergic therapy, we evaluated the prevalence of dyskinesias and motor fluctuations in PD patients using the UPDRS Part IV [14]. Patients were divided into tremor dominant (TD) or postural instability and gait dysfunction dominant (PIGD) subtype, or an intermediate subtype, according to previously described methods [15]. Activities-specific Balance Confidence scale (ABC), the standard “Timed UP-and-Go” (TUG) test and the Freezing of Gait Questionnaire (FOGQ) were used to assess the functional balance and balance confidence to predict the fall risk in people with PD. The ABC [16] was a popular, valid and reliable tool consisting of 16 items with a total score that ranges between 0 and 100, where higher score was indicative of higher balance confidence. The TUG test [17] was a commonly used assessment in older people that involves observing and timing the participant while they rised from an armchair, walked three meters, turned, walked back and sit down. A time of 13.5 seconds or longer to complete the test was considered significant in older adults for discriminating fallers from non-fallers [18]. The FOG-Q was used to assess self-perception of FOG [19]. Patients were divided into “freezers” (FOG-Q item-3 ≥1) or “non-freezers” (FOG-Q item-3 = 0) based on FOG-Q item 3 (“*Do you feel that your feet get glued to the floor while walking*, *making a turn or when trying to initiate walking?”*) [20]. The Parkinson’s disease sleep scale (PDSS) [21] and Epworth Sleep Scale (ESS) [22] were used to investigate the quality of sleep in PD patients. The PDSS was a visual analogue scale addressing 15 commonly reported symptoms associated with sleep disturbance in Parkinson’s disease that ranges from 0 to 10, where higher score was indicative of better sleep state [21]. The maximum cumulative score for the PDSS was 150 [21]. Scores of 105 or higher suggested normal sleep, and scores of 90 or less suggested the presence of sleep disturbances [23]. ESS was a simple, self-administered questionnaire intended to measure the subject’s general level of daytime sleepiness. Subjects with an ESS score >10 were considered to have excessive daytime sleepiness [22]. All the participants were evaluated with the rapid eye movement (REM) sleep behavior disorder screening questionnaire (RBDSQ) [24] and a score of at least six positive answers was proposed as the cut-off value for clinical probable RBD [25]. The Fatigue Severity Scale (FSS) was used to evaluate the severity of fatigue symptoms and a score of 36 or more was indicative of fatigue [26, 27]. The 39 item Parkinson’s disease questionnaire (PDQ-39), a 39-item and 8-dimension self-completed questionnaire was used to assess the health-related quality of life in people with PD and the Parkinson’s disease summary index (PDSI) could provide an indication of the global impact of Parkinson’s disease on health status [28]. Odor discrimination was performed with the 16-item odor identification test from extended version of sniffin’ sticks (SS-16; BurghartMesstechnik, Wedel, Germany) as previously described [29] and the cut-off score of SS-16 was 9.5 [30]. The Mini-Mental State Examination (MMSE) [31] was used to evaluate cognitive function. The Hamilton anxiety scale (HAMA) [32] and Hamilton depression scale (HAMD) [33] were used to evaluate the severity of anxiety and depressive symptoms, respectively.

### Statistical analysis

All statistical analyses were performed using the SPSS 16.0 software for Windows (SPSS Inc., Chicago, IL, USA). Demographic and disease-related characteristics were summarized with descriptive statistics. The T test, Mann-Whitney U test and chi-square test were used for comparison between groups, when appropriate.

Spearman rank correlation coefficients were calculated to assess the association between explanatory variables and QoL.

All significant (*p*<0.05) univariately associated explanatory variables were stepwise entered linear multiple regression models with QoL as dependent variable. All statistical tests were two-tailed, and the limit of significance was set at a level of 0.05.

## RESULTS

**Figure 1.**
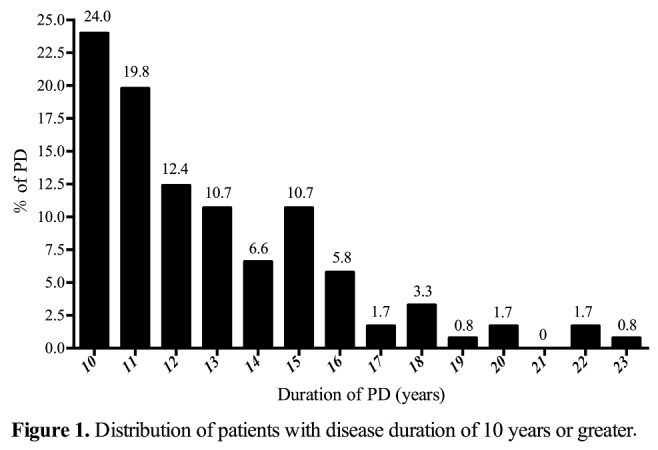
Distribution of patients with disease duration of 10 years or greater.

The entire PD-10 population had disease duration ranging from 10 to 23 years since symptom onset and the mean duration was 12.8 years. 84.2% of the total cohort was found skewed to between 10 and 15 years’ duration (Figure 1). The demographic and clinical characteristics were shown in Table 1. The mean age of these patients (55.37% male) was 66.46±8.57 years. Approximately 60.33% had motor signs lateralized predominantly to the left side of body. The incidence of motor fluctuations and dyskinesia was 64.46% and 23.14%, respectively. Approximately two thirds (64.46%) were likely to present PIGD phenotype and almost similar prevalence (61.98%) of FOG. They seemed not to struggle with sleep problems such as insomnia or daytime sleepiness other than RBD. More than half (56.20%, 68/121) of patients were likely to present RBD and nearly nine-in-ten (88.43%, 107/121) of patients had a diminished sense of smell. These PD-10 patients also experienced anxiety and depression. It was found that a high mean daily levodopa equivalent dose of dopaminergic drugs (954.52 ± 359.69 mg) had been used in the PD-10 patients.

**Table 1:** Demographic and motor characteristics for PD patients with 10 years or more disease duration.

	Overall	H-Y ≥ 3.0 n=53	H-Y < 3.0 n=68	*P*
Age (years)	66.46 ± 8.57	67.25 ± 8.64	65.84 ± 8.52	0.37
Age at onset (years)	53.70 ± 8.53	54.21 ± 8.49	53.31 ± 8.61	0.57
Sex, Male, n (%)	67 (55.37)	25 (47.17)	42 (61.76)	0.11
Disease duration (years)	12.84 ± 2.92	13.25 ± 2.87	12.53 ± 2.93	0.07
Asymmetry (left), n (%)	73 (60.33)	33 (62.26)	40 (58.82)	0.70
Hoehn &Yahr stage	2.70 ± 0.77	3.40 ± 0.57	2.16 ± 0.35	<0.01*
UPDRS-I score	3.95 ± 2.42	4.85 ± 2.54	3.25 ± 2.08	<0.01*
UPDRS-II score	17.17 ± 6.25	20.15 ± 5.96	14.84 ± 5.47	<0.01*
UPDRS-III score	34.31 ± 11.89	38.66 ± 11.71	30.93 ± 10.97	<0.01*
Motor fluctuations, n (%)	78 (64.46)	39 (73.58)	39 (57.35)	0.06
Dyskinesia, n (%)	28 (23.14)	13 (24.53)	15 (22.06)	0.75
TD phenotype, n (%)	26 (21.49)	4 (7.55)	22 (32.35)	<0.01*
PIGD phenotype, n (%)	78 (64.46)	39 (73.58)	39 (57.35)	0.06
Intermediate, n (%)	17 (14.05)	10 (18.87)	7 (10.29)	0.18
FOG, n (%)	75 (61.98)	44 (83.02)	31 (45.59)	<0.01*
ABC (%)	64.40 ± 26.64	50.25 ± 27.99	75.42 ± 19.52	<0.01*
Standardized TUG	19.98 ± 21.88	28.82 ± 30.67	13.47 ± 6.83	<0.01*
LED (mg)	954.52 ± 359.69	1033.40 ± 326.46	893.05 ± 374.50	0.03*
PDSS	98.79 ± 26.83	92.64 ± 26.24	103.59 ± 26.48	0.03*
ESS	8.40 ± 6.24	8.21 ± 5.75	8.54 ± 6.63	0.77
SS-16	5.42 ± 3.19	4.91 ± 3.36	5.82 ± 3.02	0.12
RBDSQ	5.87 ± 2.66	6.06 ± 2.53	5.72 ± 2.77	0.49
HAMA	14.48 ± 9.28	16.66 ± 9.74	12.78 ± 8.59	0.02*
HAMD	10.79 ± 6.20	13.04 ± 6.46	9.03 ± 5.42	<0.01*
FSS	42.32 ± 18.01	46.21 ± 16.62	39.29 ± 18.57	0.03*
MMSE	26.11 ± 4.00	25.74 ± 4.00	26.40 ± 4.00	0.15

**Table 2:** showed the frequency distribution of medication in PD-10 patients. Although 100% patients were taking levodopa, only 9.09% (11/121) of those were taking levodopa alone and the majority of those were also taking dopamine agonists or any other PD medication. Among PD-10 patients, 43.80% (53/121) of patients were taking three PD medication, 83.02% (44/53) of whom were taking the combination of dopamine agonists, levodopa and other anti-parkinson drugs. Although higher percentage of PD-10 patients with H-Y ≥3.0 was taking COMT inhibitor (39.62% vs. 19.12%), the percentages of taking amantadine (28.30% vs. 48.53%) for patients with H-Y ≥3.0 were significantly lower. We did not observe statistically significant differences in the proportions of levodopa, dopamine agonist, MAO-B inhibitor, anticholinergics and types of PD medication used between PD-10 patients with and without H-Y ≥3.0.

Mean scores of PDQ-39 domains were presented in Table 3. In the PD patients with 10 years or more survival, overall quality of life was reduced in all domains and disease burden was increased. The mean scores of PDQ-39 SI was 29.98 ± 17.80. Cognition (mean score 37.96 ± 23.48) was the most negative quality of life domain, mobility (mean score 34.94 ± 27.85) was the second most negative domain, however, social support (mean score 12.81 ± 22.18) was the least negative domain.

As is well known that H-Y stage was an important predictor of clinical outcome events. Patients were stratified into 2 groups [34]: H-Y score of 2.5 or less (n=68) or H-Y score of 3.0 or higher (n=53). As shown in Table 1, comparison between PD-10 patients with H-Y≥3.0 and patients with H-Y<3.0 revealed significant association of H-Y stage with UPDRS-I score (4.85 ± 2.54 vs. 3.25 ± 2.08 years, *p*<0.01), UPDRS-II score (20.15 ± 5.96 vs.14.84 ± 5.47 years, p<0.01), UPDRS-III score (38.66 ± 11.71 vs. 30.93 ± 10.97 years, p<0.01), TD phenotype (7.55% vs. 32.35%, *p*<0.01), FOG (83.02% vs. 45.59%, *p*<0.01), ABC score (50.25 ± 27.99 % vs.75.42 ± 19.52 %, *p*<0.01), TUG score (28.82 ± 30.67 vs. 13.47 ± 6.83 seconds, *p*<0.01), PDSS score (92.64 ± 26.24 vs. 103.59±26.48, *p*=0.03), LED (1033.40 ± 326.46 vs. 893.05 ± 374.50, *p*=0.03), FSS score (46.21 ± 16.62 vs. 39.29 ± 18.57, *p*=0.03), HAMA score (16.66 ± 9.74 vs. 12.78 ± 8.59, *p*=0.02) and HAMD score (13.04±6.46 vs.9.03 ± 5.42, *p*<0.01). In addition, there was significant difference in PDQ-39 summary index (37.72 ± 18.96 vs. 23.94 ± 14.28%, *p*<0.01), including mobility (50.19 ± 27.39 vs. 23.05 ± 21.90 %, *p*<0.01), activity of daily life (43.71 ± 22.0 vs. 24.14±21.04%, *p*<0.01), emotional well-being (39.86 ± 29.40 vs. 26.84 ± 23.82, p=0.01), social support (19.81 ± 27.99 vs. 7.35 ± 14.29, p=0.01), and pain (42.77± 24.02 vs. 27.45 ± 27.40, *p*<0.01) domains (Table 3). Although higher percentages of PD-10 patients with H-Y ≥3.0 had motor fluctuations (73.58% vs. 57.35%), no significant difference was found between the two groups.

**Table 2:** PD medication of H-Y ≥ 3.0 and H-Y < 3.0 patients.

	Overall	H-Y ≥ 3.0 n=53	H-Y < 3.0 n=68	*P*
Levodopa	121 (100%)	53 (100%)	68 (100%)	-
Dopamine agonist	79 (65.29%)	30 (56.60%)	49 (72.06%)	0.08
MAO-B inhibitor	33 (27.27%)	16 (30.19%)	17 (25.00%)	0.53
COMT inhibitor	34 (28.10%)	21 (39.62%)	13 (19.12%)	0.01*
Amantadine	48 (39.67%)	15 (28.30%)	33 (48.53%)	0.02*
Anticholinergics	26 (21.49%)	9 (16.98%)	17 (25.0%)	0.29
One PD medication alone (Levodopa alone)	11 (9.09%)	7 (13.21%)	4 (5.88%)	0.21
Two PD medication	32 (26.45%)	14 (26.42%)	18 (26.47%)	0.99
Three PD medication	53 (43.80%)	22 (41.51%)	31 (45.59%)	0.65
Four PD medication	19 (15.70%)	7 (13.21%)	12 (17.65%)	0.51
Five PD medication	6 (4.96%)	3 (5.66%)	3 (4.41%)	1.00

Disease duration, UPDRS Parts I-III, H-Y stage, FOG, ABC, TUG, PDSS, ESS, FSS, LED, RBDSQ, HAMA, HAMD, SCOPA-AUT and MMSE were identified as explanatory factors of QoL in the PD-10 patients. Stepwise linear multiple regression analysis was performed on all the explanatory factors and the final model (Table 4) indicated the PDSI was primarily associated with mood symptoms and self-evaluation of balance confidence and activities of daily life. Increasing excessive daytime sleepiness and disease duration contributed additionally to reduced QoL. The full model explained 75% of the variance in the PDSI.

## DISCUSSION

We explored 121 patients with disease duration of at least 10 years (PD-10) and the majority was between 10 and 15 years’ duration. The data showed that, on average, the majority of patients were in their sixth decade and had motor signs lateralized predominantly to the left side of body. Increasing motor disability appeared to be compounded by increased prevalence of non-motor symptoms and reduced quality of life, which is in accordance with other studies [1-3]. The results of the analysis grouped by H-Y stage (H-Y ≥3.0 vs. H-Y<3.0) revealed that the more advanced stage of disease in PD-10 patients was associated with motor phenotype, FOG, higher UPDRS sub-scores and levodopa equivalent doses, less balanced confidence, reduced quality of life, worse TUG performance, anxiety and depression. Self-reported mood symptoms, decreased balance confidence and reduced daily activities were the factors most closely associated with poorer QoL, but excessive daytime sleepiness and long disease duration additionally contributed to reduced QoL.

**Table 3:** PDQ-39 scores of H-Y ≥ 3.0 and H-Y < 3.0 patients.

	Overall	H-Y ≥ 3.0 n=53	H-Y < 3.0 n=68	*P*
PDQ-39 mobility (%)	34.94 ± 27.85	50.19 ± 27.39	23.05 ± 21.90	<0.01*
PDQ-39 ADL (%)	32.71 ± 23.49	43.71 ± 22.00	24.14 ± 21.04	<0.01*
PDQ-39 emotion (%)	32.54 ± 27.08	39.86 ± 29.40	26.84 ± 23.82	0.01*
PDQ-39 stigma (%)	29.49 ± 28.17	32.67 ± 27.75	27.02 ± 28.46	0.20
PDQ-39 social support (%)	12.81 ± 22.18	19.81 ± 27.99	7.35 ± 14.29	0.01*
PDQ-39 cognition (%)	37.96 ± 23.48	42.10 ± 24.58	34.74 ± 22.25	0.09
PDQ-39 communication (%)	25.21 ± 25.13	30.66 ± 27.92	20.96 ± 22.00	0.06
PDQ-39 pain (%)	34.16 ± 26.97	42.77 ± 24.02	27.45 ± 27.40	<0.01*
PDQ-39 summary index (%)	29.98 ± 17.80	37.72 ± 18.96	23.94 ± 14.28	<0.01*

**Table 4:** Factors associated with QoL in stepwise multiple linear regression model.

Model	Unstandardized Coefficients		Standardized Coefficients	*t*	*P*

	B	Std. Error	Beta		
HAMA	0.643	0.125	0.339	5.148	<0.001
ABC	-0.175	0.037	- 0.254	-4.703	<0.001
UPDRS-II score	0.665	0.154	0.225	4.310	<0.001
ESS	0.562	0.139	0.196	4.044	<0.001
HAMD	0.632	0.189	0.222	3.344	0.001
Disease duration	0.716	0.296	0.116	2.417	0.017
Adjusted R^2^	0.753				

Some researchers reported that women had a lower adjusted risk of death than men in PD [35, 36]. However, similar to previous studies [3, 37], our data did not reflect this characteristic. Our study included 67 males and 54 females, and the male to female ratio was approximately 1.2:1.0. This discrepancy, consistent with the controversy [38-40], whether gender could be considered as a potential factor, could be due to small sample size and racial differences. It was worth noting that the proportion of male patients in this Chinese population was lower (55.37 % male vs. 44.63% female; ratio 1.2:1.0) than the reported overall PD sex ratio of 1.5-2.0 times higher in males [41, 42]. Munhoz *et al* [5] reported that left-sided onset was associated with long disease duration and ambulatory PD survival. Among 121 subjects screened for laterality, approximately two thirds (60.33%) had left-sided onset. It is well known that the motor symptoms of PD are mainly due to progressive asymmetric degeneration of nigral dopaminergic neurons. However, the pathology for the lateralization in the rate of degeneration of nigral dopaminergic neurons in PD remains unclear.

In accordance with previous reports [2, 3], we found that PD-10 patients were likely to manifest the PIGD motor phenotype, which was commonly associated with an increased mortality risk and greater functional disability [2, 15]. Our data showed that PD-10 patients had low ABC score, bad TUG test performance, and high prevalence of FOG. Motor complications, which were closely linked to L-dopa dose [4], were also identified as common symptoms in PD-10 patients. Our results identified and confirmed bad mobility performance, fast disease progression and poor QoL in the long-term PD survivors. The results of the PDQ-39 domains were concordant, as mobility discomfort affected the PD-10 patients greatly, which was in accordance with the study by Kadastik-Eerme et al [43]. In comparison with mobility, cognition was found to be more negative quality of life domain in a cohort of relatively longer duration PD. The prevalence of dementia would dramatically increase after 10 years, as suggested by Reid et al. [44] who demonstrated the evolution of dementia within PD was associated with the age, regardless of the time of PD onset and the stage of disease. Although the PD-10 patients were not likely to suffer from cognitive impairment according to mean MMSE score and prevalence of cognitive decline in this study, more sensitive examinations, such as Frontal Assessment Battery, Stroop test, Verbal Digit Span forward and backward, Phonological verbal fluency would be used to detect cognitive function in PD-10 patients.

To our knowledge, there is little report of an association between long disease duration and non-motor symptoms in PD patients. This study suggested that PD-10 patients were more likely to present RBD, fatigue, anxiety and depression. Moreover, higher HAMA, HAMD, FSS, PDSS scores were associated with advanced H-Y stage. Despite the reported strong correlation of distressing fatigue with depression, anxiety and sleep disturbances [45, 46], most studies agree that all four symptoms are independent factors [47]. There is currently much interest in whether they share pathophysiologic mechanisms.

We found significant differences between H-Y stage groups in mobility, balance, motor phenotype, quality of life, fatigue, sleep problems, LED and mental problems; however, we did not find the difference between disease stage groups was significant for age, age at onset, gender and laterality in this study. As has been reported [1-3], patients with PD-10 in general, but particularly in patients with H-Y score ≥3.0, were unable to move around confidently, keep mental health and enjoy better life quality.

As hypothesized, mood problems would contribute more to reduced QoL than other features of PD, consistent with recent literature [48-50]. In our study, subjective complaints about mood symptoms, as measured with the HAMA and HAMD, accounted for the largest proportion of variance in the PDSI. Along with mood complaints, decreased balance confidence and reduced daily activities were the factors most closely associated with poorer QoL, but excessive daytime sleepiness and long disease duration were identified as important determinants of poor QoL, which was in accordance with other studies [50-53]. Our findings were consistent with previous reports showing that non-motor symptoms including depression, anxiety, sleep dysfunction may play a more important role than motor symptoms in PD patients’ QoL [43, 49, 54].

There are another two issues need to be addressed. Firstly, our study is cross-sectional and descriptive in nature. This is similar to other reports that have attempted to investigate this issue [3, 5, 6]. The confirmation of the interconnected relationship of QoL and clinical events in long duration PD patients would require a long-term large sample randomized prospective trial. Secondly, we evaluated all the PD-10 patients during the on state. With increasing motor disability, it was significantly difficult for PD patients with 10 years or greater to come to hospital during the off state. This study suggested that, during the on state, PD-10 patients tended to present increased motor and non-motor symptoms and reduced QoL. Not to mention the worse QoL and more serious symptoms PD-10 patients would suffer from during the off state. It is of great significance to identify the main issues facing PD-10 patients, and to determine factors that contribute to QoL.

In conclusion, this observational study suggested that a large proportion of PD-10 patients are suffering from both increasing clinical symptoms and worsening of quality of life. Self-report indices of mood status, balance confidence and daily activities were identified as the main determinants of poor quality of life (QoL). Improvement in access to health care quality and in tools to relieve the disease burden is an issue of crucial importance to these long duration PD patients.
